# Vesicocutaneous fistula formation during treatment with sunitinib malate: Case report

**DOI:** 10.1186/1471-230X-10-128

**Published:** 2010-11-01

**Authors:** Koichiro Watanabe, Satoshi Otsu, Ryotaro Morinaga, Sakura Kawano, Yoshinori Hirashima, Hiroyuki Sakashita, Kuniaki Shirao

**Affiliations:** 1 Department of Medical Oncology, Oita University Faculty of Medicine 1-1 Idaigaoka, Hasama-machi, Yufu-shi, Oita 879-5593, Japan; 2 Department of Integrated Pulmonology, Tokyo Medical and Dental University 1-5-45 Yushima, Bunkyo-ku, Tokyo 113-8510, Japan

## Abstract

**Background:**

The oral multi-kinase inhibitor sunitinib malate improves the survival of patients with gastrointestinal stromal tumors (GIST) after the disease progresses or intolerance to imatinib mesylate develops. Urinary fistulae arising during treatment with sunitinib for GIST have not been described.

**Case presentation:**

We describe a 62-year-old female patient diagnosed with unresectable GIST that involved the abdominal wall, urinary bladder wall, bowel, mesentery and peritoneum in the pelvic cavity. Intestinocutaneous fistulae developed on a surgical lesion after orally administered imatinib was supplemented by an arterial infusion of 5-flurouracil. Sunitinib was started after the patient developed resistance to imatinib. On day 4 of the fourth course of sunitinib, a widely dilated cutaneous fistula discharged large amounts of fluid accompanied by severe abdominal pain. Urinary communication was indicated based on the results of an intravenous injection of indigo carmine. Computed tomography findings suggested a small opening on the anterior urinary bladder wall and fistulous communication between the bladder and abdominal walls bridged by a subcutaneous cavity. The fistula closed and the amount of discharge decreased when sunitinib was discontinued. Therefore, sunitinib might have been associated with the development of the vesicocutaneous fistula in our patient.

**Conclusion:**

This is the first description of a vesicocutaneous fistula forming while under sunitinib treatment. Clinicians should be aware of the possible complication of vesicocutaneous fistula formation during treatment with molecular targeting agents in patients with extravesical invasion and peritoneal dissemination of GIST.

## Background

Sunitinib malate is a multi-kinase inhibitor of platelet-derived growth factor receptors (PDGFRα, PDGRFβ), vascular endothelial growth factor receptors (VEGFR1, VEGFR2, VEGFR3), stem cell factor receptor (KIT), Fms-like tyrosine kinase-3 (FLT3), colony-stimulating factor receptor Type 1 (CSR-1R), and the neurotrophic factor receptor (RET) derivative of a glial cell line [1]. Sunitinib has improved clinical outcomes for patients with progressive unresectable gastrointestinal stromal tumors (GIST) or who develop intolerance to imatinib mesylate and it is reasonably well tolerated [1-5]. A Phase III GIST study found objective response rates in sunitinib and placebo groups of 7% and 0%, respectively (p = 0.006) [6]. Only 9% of patients discontinued sunitinib therapy due to adverse events compared with 8% in the control group. The most common adverse reactions that occurred in over 20% of patients were fatigue (34%), diarrhea (29%), skin discoloration (25%), nausea (24%) and anorexia (19%), and all were typically of mild to moderate intensity.

An adjacent organ is usually displaced by GIST. Fistula formation is rare in patients with untreated GIST and in those treated with sunitinib for intra-abdominal malignancies [7]. We describe the clinical aspects of a rare vesicocutaneous fistula that was associated with sunitinib therapy.

## Case presentation

A 62-year-old woman was diagnosed with c-kit-positive GIST of the ascending colon with a single hepatic lesion. She was surgically treated by right hemicolectomy and partial hepatectomy. A histological assessment of a specimen of the hepatic lesion revealed GIST metastasis. Therefore, imatinib mesylate (400 mg/day) was started. After 2 years of imatinib treatment, the disease recurred in the liver, mesentery and peritoneum. Increasing the dose of imatinib to 600 mg/day did not prevent disease progression, so an arterial infusion of 5-fluorouracil (5-FU) was added to the imatinib treatment for 3 months. Thereafter, intestinocutaneous fistulae developed on the previous surgical wound and she was referred to our hospital for further treatment for recurrence.

**Figure 1:**
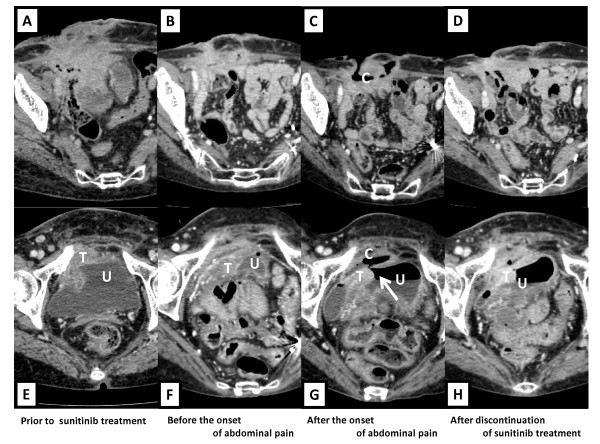
**CT findings**. U, urinary bladder; T, tumor (GIST); C, subcutaneous cavity. Tumor was located between abdominal wall (A) and urinary bladder (E) before sunitinib therapy. Abdominal tumor was smaller on day 4 of fourth course of sunitinib (B and F). Vesicocutaneous fistula bridged by subcutaneous cavity at onset of abdominal pain (C and G). Small opening (arrow) at anterior bladder wall. Air collected in urinary bladder. Fistula healed after sunitinib discontinuation (D and H) and subcutaneous cavity became smaller.

Her baseline performance status was excellent. A physical examination revealed right-sided abdominal tenderness. A small amount of a fetid discharge exuded from the cutaneous fistulae, suggesting communication with the intestine. An ostomy bag was therefore placed around the fistulae. Contrast-enhanced computed tomography (CT) images revealed that an abdominal mass involved the abdominal wall, urinary bladder wall, bowel, mesentery and peritoneum in the pelvic cavity (Figure 1A and 1E) and that a 1.3-cm low-density lesion was located in the right lobe of the liver. Signs of active infection had been absent.

Sunitinib was initiated as a standard regimen (50 mg/day for 4 weeks, every 6 weeks) for the peritoneal and liver metastases. The patient developed mild diarrhea (NCI Common Terminology Criteria for Adverse Events; grade 2) accompanied by mild abdominal pain, fever and leukocytosis on day 11 of the first course of sunitinib. No obvious signs of infection sign were evident on CT images or in culture samples of urine, stool, blood and sputum. Sunitinib was re-administered at a dose of 37.5 mg/day. The patient developed grade 2 hypothyroidism on day 8 and grade 3 vomiting with severe dehydration on the day 18 of the second course. Therefore, sunitinib was discontinued until recovery. During the third course of sunitinib, the patient tolerated a reduced dose of 25 mg/day, despite the presence of grade 1 non-hematological toxicities such as hand-foot syndrome, nausea and diarrhea. At the end of the third course, she had frequent urination with micro-hematuria. Leukocytopenia and elevated C-reactive protein were not found. The symptoms disappeared during antibiotic therapy.

**Figure 2:**
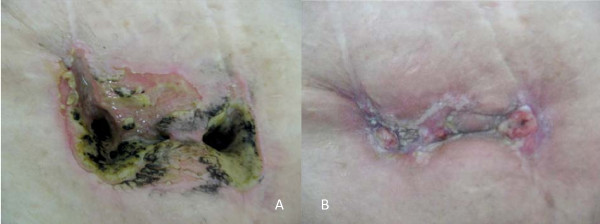
**Gross findings on day 4 of fourth course of sunitinib (A) and after its discontinuation (B)**. Cutaneous fistula orifice closed after sunitinib discontinuation.

On day 4 of the fourth course of sunitinib, the abdominal mass was reduced on CT images (Figure 1B and 1F) and the hepatic mass decreased in density but not in size. Immediately after this CT examination, the patient developed severe abdominal pain with moderate muscle defense. A large amount of fluid material was concomitantly discharged from the cutaneous fistulae. Urinary communication was indicated from the results of an intravenous indigo carmine injection. The fistula orifice was markedly dilated on CT images (Figure 1C and Figure 2A) and a small opening in the anterior bladder wall was evident (Figure 1G). Urinary leakage was localized in the subcutaneous cavity and pan-peritonitis was not evident. The abdominal symptoms gradually improved with decreased fluid drainage after sunitinib was discontinued. One month later, the size of the fistula had also obviously decreased (Figure 1D and 1H) and the fistula orifice was closed (Figure 2B). The clinical course indicated that sunitinib might have been associated with formation of the vesicocutaneous fistula in our patient.

## Discussion

Sunitinib is reasonably well tolerated and patients with GIST seldom develop fistulae while under treatment with this drug [3,6]. The GIST in our patient involved a cutaneous fistula on a previous surgical wound and another on the urinary bladder wall. Vesicocutaneous fistulae developed while she was under treatment with sunitinib, and healed upon discontinuing the drug. These findings indicated that sunitinib might be associated with the fistula formation.

Sunitinib reduced the tumor at the onset of vesicocutaneous fistula formation (Figure 1B and 1F). A multi-centre phase II study (NCIC CTG Trial IND.184) evaluated the activity of sunitinib in 19 women with locally advanced or metastatic cervical carcinoma [8]. The patients enrolled in that study, including four who developed fistulae, did not achieve objective responses. On the other hand, notable tumor shrinkage resulted in the complete healing of a fistula in a patient with GIST under imatinib therapy [9]. Taken together, tumor reduction alone might not be sufficient for fistula formation and other mechanisms are suggested.

Sunitinib has potent anti-angiogenic effects and it exerts anti-tumor activities by inhibiting blood vessel growth via inhibition of the VEGF-VEGFR pathway [5,6]. These anti-angiogenic properties play a critical role in impaired mucosal homeostasis and wound healing. Sunitinib causes tumors to shrink and tumor cell necrosis results as a consequence of a decrease in the number of vessels and reduced blood flow in the center of tumors [6,10]. Urinary leakage in our patient was localized to the cavity that formed between a potentially fragile urinary bladder wall and the part of the abdominal wall that involved GIST. Rapid degradation in the center of a tumor contrasting with a sustained rim of well-vascularized tumor tissue in the surrounding area can form a pseudo-capsule at the interface between a tumor and normal tissue [6].

## Conclusions

This is the first description of a vesicocutaneous fistula forming while under sunitinib treatment in a patient with GIST. The anti-tumor effect of the drug might be associated in part with the development of vesicocutaneous fistulae. The tumor involved the abdominal wall, urinary bladder wall, bowel, mesentery and peritoneum in the pelvic cavity. Clinicians should be aware of the possibility of vesicocutaneous fistula formation in patients with peritoneal metastasis of GIST during treatment with molecular targeting agents.

## Consent

Written informed consent was obtained from the patient to publish this case report and accompanying images. A copy of the written consent is available for review by the Editor-in-Chief of this journal.

## Abbreviations

CT: computed tomography; NCIC CTG: National Cancer Institute of Canada Clinical Trials Group.

## Competing interests

The authors declare that they have no competing interests.

## Authors' contributions

KW was involved in writing, reviewing, editing and finalizing the manuscript. SO and RM were involved in writing, editing and reviewing the manuscript. SK, YH and HS participated in the care of the patient and assisted in drafting the manuscript. KS was the chief oncologist involved with the case and also reviewed, edited and finalized the manuscript. All authors have read and approved the final version.

## Pre-publication history

The pre-publication history for this paper can be accessed here:

http://www.biomedcentral.com/1471-230X/10/128/prepub
